# A systematic review of the epidemiology of hepatitis E virus in Africa

**DOI:** 10.1186/1471-2334-14-308

**Published:** 2014-06-05

**Authors:** Jong-Hoon Kim, Kenrad E Nelson, Ursula Panzner, Yogita Kasture, Alain B Labrique, Thomas F Wierzba

**Affiliations:** 1 International Vaccine Institute, SNU Research Park, San 4-8, Nakseongdae-dong, Gwanak-gu, Seoul 151-919, South Korea; 2 Department of Epidemiology, Bloomberg School of Public Health, Johns Hopkins University, 615 N. Wolfe Street, Baltimore, MD 21205, USA

**Keywords:** Hepatitis E, Africa, Review, Outbreak, Pregnancy

## Abstract

**Background:**

Hepatitis E Virus (HEV) infection is a newly recognized serious threat to global public health and Africa is suspected to be among the most severely affected regions in the world. Understanding HEV epidemiology in Africa will expedite the implementation of evidence-based control policies aimed at preventing the spread of HEV including policies for the use of available resources such as HEV vaccines.

**Methods:**

Here we present a comprehensive review of HEV epidemiology in Africa based on published data. We searched for articles on HEV epidemiology in Africa from online databases such as PubMed, Scopus, and ISI Web of Science and critically reviewed appropriate publications to extract consistent findings, identify knowledge gaps, and suggest future studies.

**Results:**

Taking a particularly high toll in pregnant women and their fetuses, HEV has infected human populations in 28 of 56 African countries. Since 1979, 17 HEV outbreaks have been reported about once every other year from Africa causing a reported 35,300 cases with 650 deaths.

**Conclusions:**

In Africa, HEV infection is not new, is widespread, and the number of reported outbreaks are likely a significant underestimate. The authors suggest that this is a continent-wide public health problem that deserves the attention of local, regional and international agencies to implement control policies that can save numerous lives, especially those of pregnant women and their fetuses.

## Background

Hepatitis E virus (HEV) causes large outbreaks and sporadic cases of acute hepatitis, and an estimated one-third of the world’s population has been infected with HEV [1]. It is the most or second most common cause of acute viral hepatitis among adults throughout much of Asia, the Middle East, and Africa [2-4] although the true burden of HEV infection remains unknown [1]. HEV infection is usually self-limiting, but may develop into fulminant hepatitis with a case-fatality rate (CFR) between 1 and 2% in the general population [5], which can rise to over 40% in pregnant women, especially during the third trimester of pregnancy [6].

HEV was first identified in 1983 during the search for the causative agent of an outbreak of enterically transmitted non-A, non-B (NANB) hepatitis [7]. Its full-length genome was first cloned in 1991 [8]. It is a spherical, non-enveloped, single-stranded RNA virus belonging to the *Hepeviridae* family and the *Hepevirus* genus [9]. The existing evidence suggests all human HEV strains belong to a single serotype [10] although there are at least four genotypes (1–4) [11,12] with 24 subtypes [3,12,13]. Genotypes 1 and 2 are predominantly found in populations in developing countries whereas genotypes 3 and 4 are zoonotic and globally distributed [3]. Two recombinant HEV vaccine candidates have been clinically evaluated [14-16]. One vaccine candidate, rHEV, showed 95.5% efficacy after three doses in phase II trial in Nepalese military population while the other vaccine, HEV 239, showed 100% efficacy after three doses in phase III trial in a Chinese population. HEV 239 is licensed by China’s Ministry of Science and Technology [17], and is being produced and marketed by Xiamen Innovax Limited [18].

The first retrospectively (serologically) confirmed HEV outbreak occurred in New Delhi, India in 1955–56 with more than 29,000 symptomatic jaundiced persons [19-21]. Since then, many serologically confirmed outbreaks and sporadic cases [3,22] and probable outbreaks have occurred, especially in Asia and Africa [23]. Africa has usually been the place where resources for controlling infectious diseases are last deployed although it is among the regions most severely inflicted by infectious diseases [24]. Acknowledging that understanding HEV infection and distribution in Africa can expedite implementation of evidence-based control policies, our overall objective was to characterize the epidemiology of HEV in Africa by reviewing and summarizing relevant, peer-reviewed literature. The authors’ specific objectives were to explore rates of infection (i.e., seroprevalence, outbreaks, sporadic cases), severity (i.e., case-fatality rates), modes of transmission, and circulating genotypes. The authors also identified knowledge gaps in the existing literature and suggested future studies.

## Methods

### Searching

We searched PubMed, Scopus, and ISI Web of Science (up to March 24, 2014) using the following search terms: (“Hepatitis E” OR “Non A Non B”) AND (Country_name_1 OR Country_name_2 OR …), where ellipsis represents names of all African countries (with OR between them) as extracted from a UN list [25]. The search term includes “Non A Non B” because HEV was identified as the causative agent of the enterically transmitted NANB hepatitis [7] and thus should be responsible for at least some of NANB outbreaks. In addition, we reviewed relevant references from the articles we obtained. Articles published in English and French were included.

### Selection and methods

**Figure 1:**
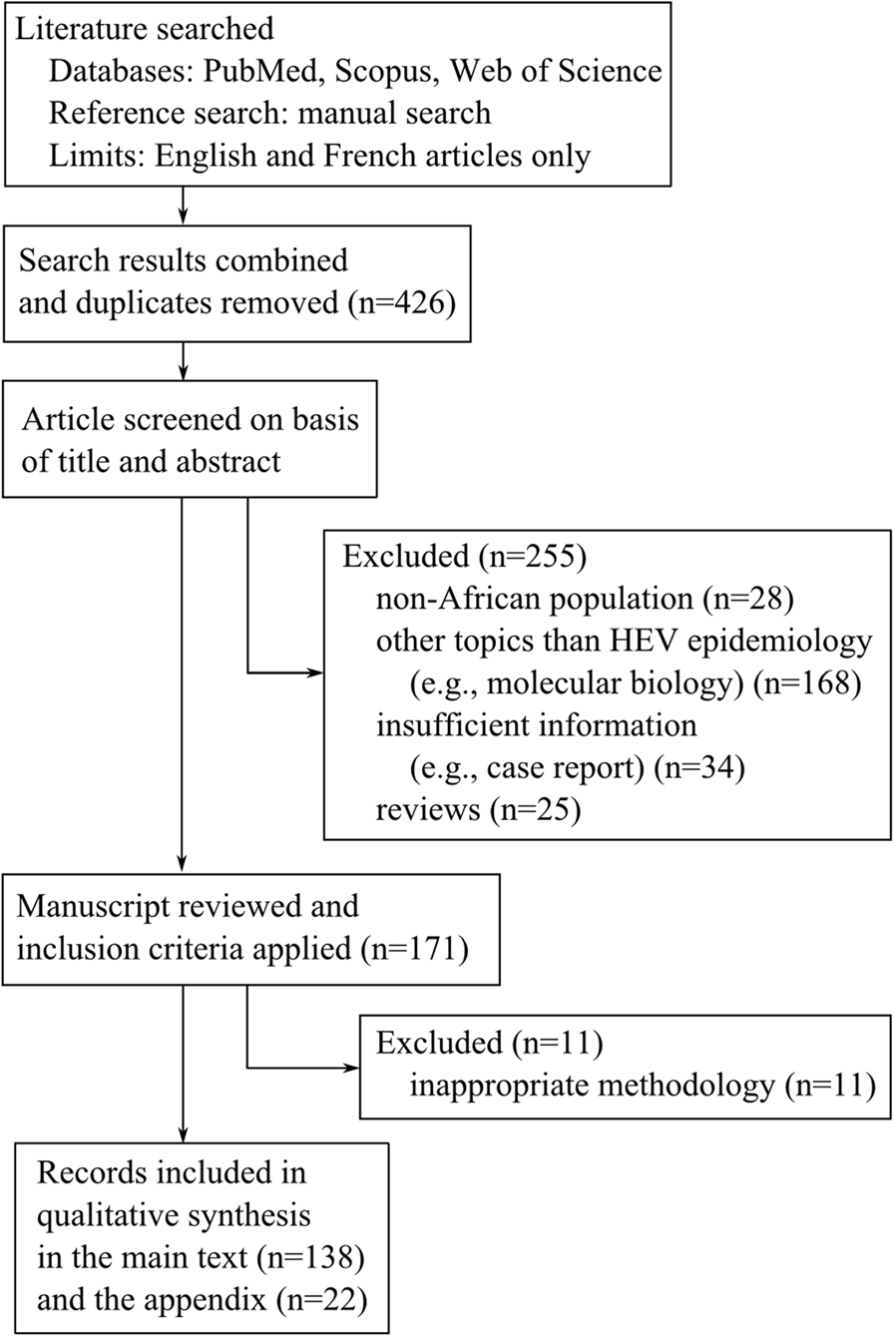
Flow diagram for study selection.

Figure 1 is a flow chart that describes the procedure of literature selection. We identified 219, 288, and 159 articles from PubMed, Scopus, and ISI Web of Science, respectively, and also reviewed articles obtained by screening references. The number of articles was 426 after removing duplicates. Of 426 articles, we synthesized 160 original research articles that provide relevant information while excluding the other 266 articles for the following reasons:

1. Non-African populations (n = 28)

2. Topics other than HEV epidemiology, e.g., molecular biology (n = 168)

3. Insufficient information, e.g., case report (n = 34)

4. Review articles (n = 25)

5. Suboptimal methodology (n = 11)

Of 160 articles, we summarized 138 articles about serologically confirmed HEV in the main text and separately summarized NANB outbreaks in the Additional file 1 (n = 22).

HEV seroprevalence analysis included articles describing serology studies for total antibodies (i.e., both IgG and IgM) or IgG to HEV by enzyme-linked immunosorbent assay, using commercial kits or in-house methods. In outbreaks, incident cases of HEV are defined by the existence of IgM antibodies to HEV or paired serum samples with a significant increase in IgG to HEV or the existence of HEV RNA measured by reverse transcriptase polymerase chain reaction. CFR was defined as the number of deaths divided by the number of laboratory confirmed cases or cases epidemiologically linked to HEV infections times 100.

### Data source

Sources of data on the epidemiology of HEV come from (i) seroprevalence studies in the general population (e.g., blood donors or healthy population) or suspected high-risk population (e.g., pig handlers or waste water treatment plant workers or pregnant women), (ii) studies of sporadic acute hepatitis patients, and (iii) investigations of hepatitis outbreaks. We also discuss studies of sporadic cases and outbreaks of NANB hepatitis viruses in the Discussion Section with tables of data in the Additional file 1. The present paper includes studies from 28 (50%) of 56 African countries: Algeria [26-28], Burkina Faso [29], Burundi [30,31], Cameroon [32], Central African Republic [33,34], Chad [27,28,35-38], Côte d’Ivoire [39], Democratic Republic of the Congo [40], Djibouti [41,42], Ethiopia [6,43], Eritrea [44], Egypt [45-71], Gabon [72,73], Ghana [32,74-78], Kenya [79,80], Madagascar [81], Mayotte [82], Morocco [83-85], Namibia [86-88], Nigeria [89-91], Senegal [92,93], Somalia [94-96], South Africa [97,98], South Sudan [99], Sudan [35,100-105], Tunisia [106-111], the United Republic of Tanzania [112-114], and Zambia [115].

## Results

### Seroprevalence of anti-HEV antibodies

Data on seroprevalence of anti-HEV antibodies comes from 35 studies in 13 African countries. Table 1 presents a summary sorted by country name and seroprevalence. Seroprevalence varies by country from 84.3% [55] among pregnant Egyptian women aged 24 years on average to 0% among village residents aged 24 years on average in Gabon [72]. The seroprevalence seems to be higher in pregnant women than in the general population in Ghana (28.7% [78] vs. 4.6% [76]) and also in Gabon (14.2% [73] vs. 0% [72]). Two studies of Ghanaians suggest that predominant HEV genotypes in that country may be of zoonotic origin: seroprevalence among pig handlers is over 34% [75,77] whereas that among general population is about 4% [76]. Seroprevalence also differs between rural and urban areas. In Gabon, one study found a higher prevalence of HEV of about 2.0 times in urban (13.5%) compared with rural areas (6.4%) [73]. On the other hand, in South Africa, HEV seroprevalence is higher in rural compared with urban residents (15.3% vs. 6.6%) [98]. The difference in seroprevalence between rural and urban residents is also observed in Egypt with seroprevalence being higher in rural areas [53].

**Table 1:** Seroprevalence of anti-HEV antibodies in Africa

**Country**	**% sero-prevalence**	**Sample demographics**	**Sample size**	**Year of sampling**	**Diagnostic methods**	**Source**
Burkina Faso	19.1	Blood donors	178	2010-12	IgG	[29]
	11.6	Pregnant women	189	2010-12	IgG	[29]
Burundi	14.0	Adults without chronic liver disease, 44.7 yrs old (±13.5)	129	1986	Total Ig	[30]
Cameroon	14.2	HIV-infected adults, 38.1 yrs old (±11.3)	289	2009-10	IgG	[32]
	2.0	HIV-infected children, 8.3 yrs old (±7.5)	100	2009-10	IgG	[32]
CAR^a^	24.2	Patients attending the center for sexually transmitted diseases	157	1995^b^	Total Ig	[33]
Djibouti	13.0	Male peacekeepers in Haiti, 31.2 yrs old	112	1998^b^	Total Ig	[42]
Egypt	84.3	Pregnant women, 24 yrs old (16–48)	2,428	1997-2003	Total Ig	[55]
	80.1	Patients with chronic liver disease, 48 yrs old (23–62)	518	2000-2	IgG	[57]
	67.6	Residents of two rural villages, 24.5 and 26.5 yrs, respectively	10,156	1997	Total Ig	[54]
	58.6	Pregnant women, ~33 yrs old	116	2009	IgG	[58]
	56.4	Residents of a semi-urban village, 1–67 yrs old	140	1993	Total Ig	[51]
	51.2	Waste water treatment plant workers, 47.1 yrs old	43	2011^b^	Total Ig	[60]
	50.6	Waste water treatment plant workers, 20–60 yrs old	233	2000^b^	Total Ig	[61]
	45.3	Blood donors, 18–45 yrs old	95	1998^b^	IgG	[52]
	54.1	Four waste water treatment plant male workers, 20–60 yrs old	205	1998-9	IgG	[116]
	39.6	Haemodialysis patients, 8–20 yrs old	96	1998^b^	IgG	[52]
	38.9	Healthy females, 21.8 yrs old (16–25)	95	1995	IgG	[50]
	17.2	Residents of a hamlet, 20.9 yrs old (<1-95)	1259	1992	IgG	[49]
	0.0	Healthy controls, 20–60 yrs old	96	1998-9	IgG	[116]
Gabon	14.2	Pregnant women, 24.6 yrs old (14–44)	840	2005, 2007	IgG	[73]
	0.0	Villagers, 29 yrs old (2–80)	35	1991-2	Total Ig	[72]
Ghana	45.3	Adult HIV patients (n = 402), 40 yrs old (±9.6)	402	2008-10	IgG	[32]
	38.1	Pig handlers, 36.5 yrs old (12–65)	105	2009^b^	Total Ig	[77]
	34.8	Pig handlers, 32.9 yrs old (15–70)	353	2008	Total Ig	[75]
	28.7	Pregnant women, 28.9 yrs old (13–42)	157	2008	Total Ig	[78]
	4.6	Blood donors	239	2012^b^	IgG	[76]
	4.4	6-18 yr olds	803	1993	Total Ig	[74]
Madagascar	14.1	Slaughterhouse workers	427	2008-9	Total Ig	[81]
Morocco	8.5	Blood donors	200	2000-1	IgG	[85]
	2.2	232 men and 259 women, 27.7 yrs old (±5.9)	491	1995^b^	IgG	[84]
Nigeria	43.0	Health care workers	88	2008-9	Total Ig	[90]
	94.0	Control healthy adults	44	2008-9	Total Ig	[90]
	13.4	Healthy and sick people, 29.8 yrs old (3–72)	186	2007	Total Ig	[91]
South Africa	10.7	Urban (n = 407) and rural (n = 360) blacks, 42.4 yrs old (18–85)	767	1996^b^	Total Ig	[98,117]
	2.6	Medical students	227	1992	Total Ig	[97]
	1.8	Canoeists who have been regularly exposed to waste water	555	1992	Total Ig	[97]
Tanzania	6.6	Women, 32.1 yrs old (15–45)	212	1996	Total Ig	[114]
	0.2	Healthy adults, 30.3 yrs old	403	1992	Total Ig	[112]
	0.0	Women, 24.5 yrs old	180	1995	Total Ig	[113]
Tunisia	46.0	Healthy persons, > 60 yrs old	100	1991	IgG	[106]
	29.5	Children with chronic haematological diseases	34	1996	IgG	[106]
	28.9	Polytransfused patients; adults (n = 59, 34.8 yrs old (20–61)) and children (n = 48, 7.3 yrs old (1–15))	107	2008-9	IgG	[107]
	22.0	Healthy blood donors, < 40 yrs old	100	1996	IgG	[106]
	12.1	Pregnant women, 30.1 yrs old (17–52)	404	2008-9	IgG	[108]
	10.0	Healthy controls; blood donors (n = 100, 31.3 yrs old (20–58)) and children, (n = 60, 7.9 yrs old (1–15))	160	2008-9	IgG	[107]
	5.4	Blood donors, 32.6 yrs old (±8.6)	687	2007-8	Total Ig	[109]
	4.3	Healthy persons, 20.7 yrs old (16–25)	1,505	2008^b^	IgG	[110]
Zambia	42	Urban adults, 18–64 yrs old	106	1999	IgG	[115]
	16	Urban children, 1–15 yrs old	194	2011	IgG	[115]

^a^CAR; Central African Republic.

^b^The year of the publication.

Seroprevalence varies by country and by subpopulation and studies were done under different conditions (e.g., sample size, demographics, and different diagnostic methods). Age of the sample is provided as mean (range or ± standard deviation, if available).

### Sporadic hepatitis cases attributed to HEV

Data on acute hepatitis E come from studies of sporadic acute hepatitis cases and are available from 29 studies in 13 African countries. Table 2 shows a summary sorted by country followed by decreasing proportion of sporadic hepatitis cases that are attributable to HEV infection. Table 2 also provides characteristics of sporadic hepatitis cases studied and types of antibodies tested (i.e., IgG or IgM). The proportion of cases that are attributable to HEV ranges from 70% in male patients aged 25–33 years in Nigeria [89] to 2% in patients aged < 15 years in Egypt [48]. It is interesting to note that existing studies show that the proportions of cases that are attributable to HEV are lower than 27% in Egypt despite their high HEV seroprevalence: the majority of acute hepatitis patients were attributable to HAV [45,48,64,70] or HBV [71]. For example, one study of acute hepatitis among Egyptian children with a mean age of 5.4 years reported that HAV was responsible for 35 out of 36 acute hepatitis cases whereas HEV was detected in only one case [48].

**Table 2:** Sporadic cases caused by hepatitis E virus in Africa

**Country**	**% sero-positivity**	**Case demographics**	**No. of cases**	**Year of sampling**	**Diagnostic methods**	**Source**
Chad	48.8	Acute or fulminant hepatitis patients, 4–64 yrs old	41	1993	IgM	[36]
	20.0^a^	Sporadic cases	17	1994	RT-PCR^b^	[27]
Djibouti	58.5	Acute hepatitis patients, 21 · 8 yrs old (2–65)	65	1992-3	IgM	[41]
Egypt	21.7	Acute hepatitis patients, 26 · 6 yrs old (18–60)	143	1993-4	IgM	[71]
	24.2	Jaundiced patients, 1–73 yrs old	202	1993	IgM	[46]
	22.2	Jaundiced children, 5 yrs old (1–11)	261	1990	IgM	[70]
	20.2	Acute viral hepatitis patients, 8 yrs old	287	2006-8	IgM	[62]
	17.9	Acute hepatitis patients, 15.7 (± 14.9) yrs old	235	2007-8	IgM or > =3-fold rise in IgG	[69]
	17.2	Children with elevated level (two-fold or more) of AST and ALT	64	2006^d^	IgM	[47]
	15.7	Acute hepatitis patients, 15.9 yrs old (1–65)	235	2007-8	IgM	[63]
	15.1	Children with acute jaundice, 6 · 4 yrs old (1–13)	73	1987-8	IgM	[45]
	12.5	Patients with acute hepatitis, 20 · 2 yrs old (4–65)	200	2001-2	IgM	[64]
	6.0	Children with minor hepatic ailments, 6 mo -10 yrs	100	2004-5	IgM	[65]
	5 · 0	Patients with acute on chronic liver failure, 46.4 yrs old	100	2009-10	IgM	[66]
	2.1	Acute viral hepatitis patients, 25 yrs old (2–77)	47	2002-5	IgM	[67]
	2.0	Hepatitis patients, 5.4 yrs old (1.5-15)	50	2007	RT-PCR	[48]
Ethiopia	45.6	Acute viral hepatitis patients with NANB	79	1988-91	FABA^d^	[43]
	31.8	Non-pregnant women with acute viral hepatitis, 30 yrs old	22	1988-91	FABA	[6]
	67 · 9	Pregnant women with acute viral hepatitis, 26 yrs old	28	1988-91	FABA	[6]
Mayotte	100.0	Patients with acute jaundice, 46 yrs old	1	2009	IgM	[82]
Nigeria	70.0	Male patients with acute hepatitis, 25–33 yrs old	10	1997-8	RT-PCR	[89]
Senegal	20.0	Patients with jaundice	30	1992^c^	IgM	[93]
	10.2	Patients with viral hepatitis	49	1993^c^	IgM	[92]
Somalia	61.1	Native Somalis and displaced Ethiopian patients with acute hepatitis, 7–90 yrs old	36	1992-3	IgM	[96]
Sudan	5.4	Patients with fulminant hepatic failure, 38 yrs old (19–75)	37	2003-4	IgM	[103]
	59.0	Children with acute clinical jaundice, ≤14 yrs old	39	1987-8	IgM	[118]

^a^20% was extrapolated from the results of RT-PCR results of 5 samples out of total 17 cases.

^b^Reverse transcription polymerase chain reaction.

^c^The year of the publication.

^d^FABA; fluorescent antibody blocking assay, which is claimed to detect acute infection, not but past infection.

Proportion of sporadic hepatitis cases attributable to HEV varies by country and by subpopulation and studies were done under different conditions (e.g., sample size, demographics, and different diagnostic methods). Age of the sample is provided as mean (range or ± standard deviation, if available).

### Outbreaks and attack rate

The first well-characterized outbreak of laboratory-confirmed Hepatitis E occurred in Côte d’Ivoire in 1986 [39]. However, assuming that hepatitis outbreaks characterized by acute jaundice and a high CFR among pregnant women were likely due to HEV, Teo identified earlier, probable HEV outbreaks in Tunisia from 1950 to 1953, Algeria from 1952 to 1956, Congo in 1958, Morocco from 1958 to 60, and Libya from 1968 to 1970 and also in 1975 [23].

HEV outbreaks have been detected in 11 African countries (17 studies), of which some outbreaks in refugee camps [79,86,100] and Table 3 presents a summary of these outbreaks sorted by country name and decreasing clinical attack rate. The highest attack rate (25.1%) of the population was observed during outbreaks in two subcounties of Kitgum District, Uganda from October 2007 to June 2009 [119,120]. Attack rate could have been even higher because the epidemic had not ended at the time of the investigation and in particular, it had not even peaked but was increasing in one of two subcounties. The lowest attack rate (2.7%) was observed during an outbreak in the Central African Republic on July 2002 [34].

**Table 3:** Hepatitis E outbreaks in Africa

**Country**	**Year**	**No. cases (deaths)**	**Clinical attack rate (population size)**	**Variance in clinical attack rates**		**Source**
				**By age**	**By gender**	
Algeria	1979-80	20	NA^a^	NA	NA	[27]
CAR^b^	Jul - Oct 2002	715	2.7%	No significant difference	No significant difference	[34]
	Jun 2004 - Sep 2005	411	NA	The age group 18–34 years was more frequently anti-HEV IgM positive (91.2%) than those aged 1–17 (78.0%) or over 34 (64.9%) (*p* < 0.001)	Risk for infection was clearly higher in males than females based on IgG seroprevalence (OR = 2.04; 95% CI 1.21-3.45; *p* < 0.005)	[121]
Chad	1983-4	34	NA	NA	NA	[27]
	Jun - Aug 2004	989 (30)	NA	NA	NA	[37,38]
Djibouti	Dec1992-Sep1993	43	NA	NA	NA	[41]
Eritrea	Oct 1988-Mar 1989	> 750	NA	81% of the patients were between 18 and 30 years of age among aged from 15 to 56.	The outbreak among military personnel; no female patients	[44]
Kenya^d^	Mar - Oct 1991	1,765 (63)	6.3% (n = 26,920)	Increased with age with a peak among those >30, while serologic attack rate is not different by age group	Clinical attack rate is 6.1% for male and 6.3% for female	[79]
	Jul - Nov 2012	349 (10)	NA	NA	184 (54.3%) were females.	[80]
Morocco	1994	> 75	NA	NA	NA	[83]
Namibia^d^	Jul - Oct 1983	201	NA	Most common in persons aged 25–29 years old among patients aged 5–54 years old	72% of 64 patients were male.	[86]
Somalia	1988 – 9, 23 months	11,759 (346)	4.7% (n = 245,312)	Increased with age groups: 5%, 13%, and 20% for those aged 0–4, 5–15, and >15 years old, respectively	Female-to-male ratio was 1.08:1	[94,95]
South Sudan	Jul 2012-Jan 2013	5,080	7.4%	Persons aged 18–59 years had the highest attack rates	NA	[99]
Sudan	Oct 1988	≥55	NA	NA	NA	[122]
	Jul - Dec 2004	2,621 (45)	3.3%^c,d^ (n = 78,800)	Being 15–45 years old was a risk factor for clinical HEV infection with odds ratio being 2.13 (95% CI, 1.02-4.46).	No significant difference	[100]
	Nov 2010-Mar 2011	39 ^e^ (11^e^)	NA	NA	Only pregnant women were reported.	[104]
Uganda	Oct 2007 -Jun 2009	>10,356 (160)	25.1% (n = 19,098)	< 2 year olds (6.9%) vs. pregnant women (87%)	22% males vs. 28% females (*p* < 0.001)	[119,120,123]

^a^NA; not available.

^b^CAR; Central African Republic.

^c^Active case finding suggested a clinical attack rate of 16%.

^d^Outbreak in refugee camps.

^e^For pregnant women.

Published reports suggest clinical HEV infection risk is highest among young adults in African populations [44,79,86,94,100,119,120] although one study reports no significant difference in the risk of clinical HEV infection by age [34]. Studies show mixed results regarding whether sex predisposes people to clinical HEV infection (i.e., higher risk for males [86] or higher risk for females [119,120] or no significant difference [34,79,94,100,123].

### Case fatality rate (CFR)

The CFR from outbreak investigations or sporadic cases were reported by 8 African countries (10 studies). Table 4 presents a summary sorted by CFR and pregnancy. CFR’s in the overall population range from 17.8% in Darfur, Sudan in 2004 [101] to 1.5% in Uganda during March to December, 2008 [123]. Among pregnant women, fatalities are considerably higher ranging from 42.1% among sporadic cases sampled during 1988-91in Ethiopia [6] to 12.5% during an outbreak in Dadaab refugee camp of Kenya in 2012 [80]. Compared with the overall population, fatalities are also higher in children under 2 years of age. One study in Uganda using active case detection and verbal autopsies found that icteric children less than two years of age experienced 13% mortality, which was higher than the 6.9% experienced among pregnant woman [120]. One author claims that the inoculum size may be important in determining CFR: CFR was 8.6% among villagers supplied by wells whereas it was 2.5% and 0.8% in those relying on river and pond water, respectively [94].

**Table 4:** Case-fatality rates (CFRs) of HEV infection

**Country**	**Year**	**Case-fatality rate (n = no. of cases)**		**Source**
		**Pregnant female**	**Overall**	
CAR^a^	2002	20% (n = 5)	1.8% (n = 222)	[34]
Chad	2004	NA^b^	3.0% (n = 989)	[37]
Eritrea	1988-89	NA	0% (n = 423)	[44]
Ethiopia	1988-91	42.1% (n = 19)	NA	[6]
Kenya	2012	12.5% (n = 72)	2.9% (n = 339)	[80]
Somalia	1988-9	13.8% (n = NA)	3.0% (n = 11,413)	[94]
South Sudan	2012-3	10.4% (n = 211)	NA	[99]
Sudan	2004	31.1% (n = 61)	17.8% (n = 253)	[101]
	2004	31.1% (n = 61)	1.7% (n = 2,621)	[100]
	2004	NA	1.7% (n = 2,472)	[37]
Uganda	2008	NA	1.5% (n = 9,648)	[123]

^a^CAR; Central African Republic.

^b^NA; not available.

### Genotype prevalence

**Figure 2:**
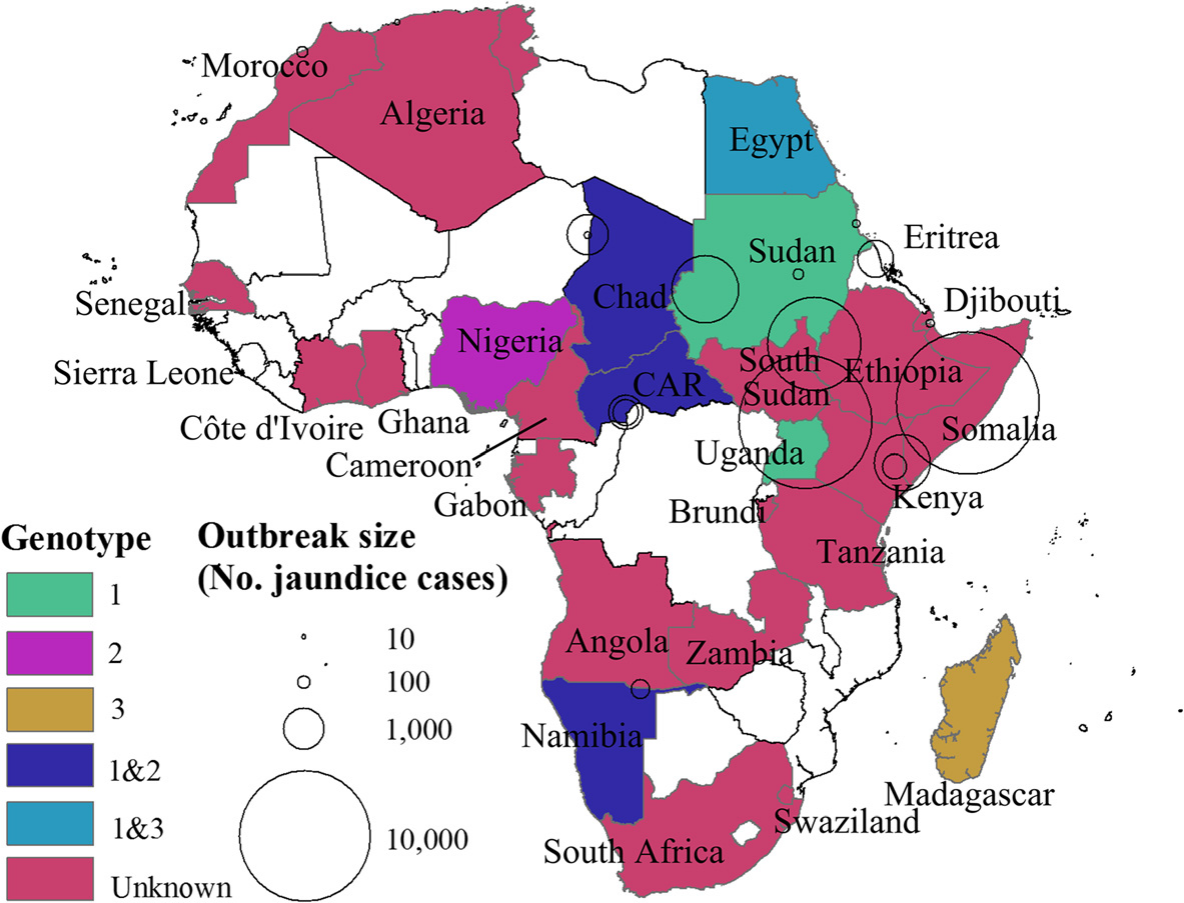
**Map of Africa.** Colored areas represent countries where HEV is endemic at least for some subpopulations or sporadic HEV cases or outbreaks have been detected. Circles indicate HEV outbreaks with centers and areas indicating the location and outbreak size, respectively. Different colors represent different genotypes. White areas indicate countries where no data is available.

Data on the genotypes of circulating HEV’s are available for 9 countries (16 studies). Table 5 presents a summary sorted by genotype and also provides characteristics of the sample, genomic regions tested. Genotype 1 seems to be most prevalent as it was found in Central African Republic [34], Sudan [35], Chad [28,35], Egypt [46,62,124], and Namibia [88] followed by genotype 2, which were observed in Central African Republic [34], Chad [35], Namibia [87], and Nigeria [6,89]. Genotype 3 is rare and was found in one Egyptian child [48], one acute hepatitis patient in Mayotte (originally from France) [82]. Genotype prevalence can differ in neighboring countries as was demonstrated by one study in Sudan and Chad where genotype 1 was more common in Sudan and genotype 2 was more common in Chad [35], Figure 2 shows a map of Africa where countries in which HEV infections were observed are differently colored according to HEV genotype.

**Table 5:** Genotype distribution from African HEVs

**Genotype**	**Country**	**Year of sampling**	**Sample**	**RNA region tested**	**Source**
1	CAR^a^	2002	One fecal sample from an outbreak	NA^b^	[34]
	Chad	1984	A patient with hepatitis E	Complete genome	[28]
		2004	Five isolates from an outbreak	ORF^c^2 (363 nt^d^)	[35]
	Egypt	1993	Acute hepatitis patients	ORF1 (location: 55–320)	[46]
		2006-8	Acute hepatitis patients	ORF1	[62]
		2012^e^	Sixteen isolates from acute hepatitis patients	ORF2 (189 nt)	[124]
	Namibia	1983	Nine isolates from an outbreak in Kavango	ORF2 (296 nt), 3 (188 nt)	[88]
	Sudan	2004	Twenty three isolates from an outbreak	ORF2 (363 nt)	[35]
	Uganda	2007	Internally displaced persons camp	NA	[123]
		2008	Twenty four isolates from an outbreak	NA	[119]
2	CAR	2002	Three fecal samples from an outbreak	NA	[34]
	Chad	2004	Four isolates from an outbreak	ORF2 (363 nt)	[35]
	Namibia	1995	Four isolates from NANB outbreak in Rundu	ORF2 (451 nt near 3’-end)	[87]
	Nigeria	2000^e^	Ten adult acute hepatitis patients	ORF1, 2 (3’-end)	[89]
3	Egypt	2007	One 9 year-old acute hepatitis patient	ORF1, 2, 2/3	[48]
	Mayotte	2009	One French acute hepatitis patient (46 yr old)	ORF2 (288 nt)	[82]
	Madagascar	2008-9	Slaughter house workers	ORF2,3 (1000 nt)	[81]

^a^CAR; Central African Republic.

^b^NA; not available.

^c^ORF; open reading frame.

^d^nt; nucleotides.

^e^Publication year.

### Mode of transmission

Studies stress the importance of fecal contamination of water as a main source of infection in outbreaks across African countries [34,44,86,94,100,101,123]. However, in a laboratory-confirmed HEV outbreak of 3,218 cases in northern Uganda, the authors suggest that some patients were infected through person-to-person transmission, which is supported by three observations: (1) HEV was not detected from water or zoonotic sources, (2) the epidemic curve suggested a propagated, not point-source outbreak, and (3) there was evidence of close contact between incident cases and subsequently infected household contacts after an incubation period [120]. Non-human reservoirs have been identified in donkeys in Sudan (unknown genotype) [100] and pigs the Democratic Republic of the Congo and Uganda (both genotype 3), and cows, buffaloes, sheep and goats in Egypt [40,120]. In Ghana, the high prevalence (34.8%) of antibodies to HEV in pig farm workers was reported with the greatest risk factor being close contact with piped-water [75,77]. No data have been reported from Africa suggesting the transmission of HEV by transfusion or by sexual intercourse [125] although the transmission of HEV by transfusion has been well documented in Europe and Japan [126,127]. A single Egyptian study suggested HEV transmission from mothers to their neonates. Of nine mothers PCR positive for HEV RNA, five (55.6%) of their neonates were also PCR positive [128]. No other studies have investigated mother-to-child transmission in Africa.

### Co-infection with other infectious diseases

Three infectious diseases—hepatitis viruses other than HEV, schistosomiasis, and HIV—are known to be associated with an increased risk or severity of HEV infection from published studies. First, infection with other hepatitis viruses was positively associated with infection with HEV. One study found a high prevalence of anti-HEV antibodies in Egyptian children infected with Hepatitis B (56.7%), Hepatitis C (52%) and both Hepatitis B and C (30%) compared with patients with non-A, non-B, non-C hepatitis (7.1%) [129]. Second, one study showed that patients with schistosomiasis infection had a higher prevalence of HEV infection: seroprevalence of HEV infection was 31% in patients with *Schistosoma mansoni* whereas it was 14% among parasite-free individuals [53]. Third, studies reported discordant findings as to whether patients with HIV infection or AIDS are at increased risk for HEV infection. One study reported that anti-HEV seroprevalence among HIV/AIDS cases in Africa was similar to HIV negatives and concluded that there is no significant increased risk for HEV infection in patients with HIV positive [30]. Another study showed the seroprevalence of anti-HEV IgG antibodies was higher among HIV-1 infected women than HTLV-I infected women (7.1% vs. 5.0%) [130]. Similarly, HEV infection was significantly more common in HIV-seropositive than HIV-seronegative Zambian adults (odds ratio = 6.2, 95% CI, 2.2-17.8). [115]

## Discussion

The first documented outbreak of HEV infection likely occurred in 1950 in Tunisia [23] and since the 1980’s, when HEV diagnostic assays became available, half of all African countries including nations in north, south, east, west, and central Africa have published articles of Hepatitis E infections. Clearly, these results suggest that HEV infection is not new to Africa and implies that this is a continent-wide public health problem and a potential threat to travelers. This is supported by a recently published study on HEV in low- and middle-income countries in Asia and Africa [131]. HEV infection deserves the attention of local, regional and international agencies.

Since nations lack routine surveillance for HEV infection, it is difficult to estimate the African disease burden. However, these publications suggest that outbreaks are frequent with about one outbreak report published every other year from Africa. As only a few outbreaks are likely identified and even fewer reported in peer-reviewed journals, it is probable that outbreaks in Africa are even more common than these reports imply. To address HEV outbreak control, routine HEV surveillance and response is necessary and could be integrated with measles or poliomyelitis surveillance, which are ongoing activities in most countries.

Among HEV outbreaks, the overall case-fatality rate is comparable to that seen with other diseases such as cholera and measles signifying that these patients require equal attention. Like HEV infections among pregnant woman in Asia, the clinical prognosis is far worse for pregnant women than for men with fatalities reaching four out of 10 infected pregnant women [6]. The concerns for pregnant woman are further amplified when recognizing that HIV prevalence is high among pregnant women in sub-Saharan Africa [132] and co-infection with HIV and HEV as reported here may increase the risk of fulminant or chronic hepatitis. African men co-infected with HIV and HEV are similarly at risk of complications. While appreciating the hardship from all HEV-associated deaths, maternal deaths are the great tragedy of HEV infection likely causing serious adverse consequences for the health and well-being of surviving family members, especially children.

It has been suggested that many hepatitis outbreaks that occurred before the development of diagnostic assays for any hepatitis virus and enterically transmitted NANB hepatitis outbreaks were likely due to HEV as CFRs in pregnant women in these outbreaks were disproportionately high and attack rates were higher in young adults compared to children or adults [23]. This review supports the prior study showing that outbreaks and sporadic cases by NANB reported in Africa, many of which were not considered in the previous study, also display high CFR in pregnant women and high clinical attack rate among young adults (Tables A1-A3 in the Additional file 1).

We noted several limitations with the existing literature. First, there is no diagnostic “gold standard” for HEV infection and existing studies use different assays that vary in sensitivity and specificity adversely impacting the validity and comparability of reports. Second, studies measure the prevalence of HEV infection in dissimilar subpopulations making it difficult to compare seroprevalence. For example, it is difficult to conclude whether the overall seroprevalence of HEV infection in South Africa is higher than that in Morocco when examining studies that show seroprevalence among South Africans with a mean age of 42 years is 10.7% [98] and that among younger population in Morocco is 2.2-6.8% [84,85]. Third, studies use different criteria to define acute hepatitis. In a study of Egyptian children, acute hepatitis was defined as the acute injury to the liver, manifested by two fold or more increase in the level of aspartate aminotransferase and alanine aminotransferase [47] whereas in another study, acute hepatitis was defined as the patient whose aminotransferase level was 5 times the normal value [133]. Finally, different surveillance methods may lead to different attack rates being determined. Attack rates are exceptionally difficult to determine in un-enumerated populations, which is likely to be the case in many African nations. The limitations noted above could be corrected in future studies by developing and publishing standardized study methods including case definitions and analytical plans and by development of a generic protocol for outbreak and endemic disease investigations. For diagnosis, an enzyme-linked immunosorbent assay for HEV IgG and IgM (Beijing Wantai Biological Pharmacy Enterprise CO., LTD.) shows high sensitivity and specificity compared to other assays including molecular methods [134].

There are knowledge gaps. Serology studies suggest that many Africans are infected with HEV, but except during outbreaks, symptomatic disease is only sporadic implying that case finding is incomplete or that only a few infected cases progress to clinical disease. Pathogenesis studies are needed. While there are a growing number of published reports on the impact of HEV infection, the overall burden of hepatitis E in Africa remains unclear. For example, two studies from South Asia using verbal autopsies suggest that HEV is responsible for 10% or more of pregnancy-associated deaths [135,136]. The same investigation should also be carried out in multiple sites in Africa. HEV transmission in nations with genotypes 1 and 2 likely occurs from the fecal-oral route. However, the authors in one study on the HEV epidemic in Uganda during 2007–9 claimed that person-to-person transmission played a significant role in the propagation of HEV [120,137]. Because of the implications for control measures, this report implies that HEV transmission studies should be carried out. Given the high background rates of HEV infection, the effect of co-infections (e.g., HIV, Hepatitis B) on the severity of disease especially in pregnant women are needed. Potential risk factors for HEV infection such as co-infections or pregnancy need to be clarified. Studies of vertical transmission and blood donor screening should be funded.

As these results suggest that acute hepatitis E is fecal-orally transmitted in African countries, the ultimate control of this disease will require increased access to safe water and sanitation and improved personal hygiene. Until these measures are universal, an anti-HEV vaccine is needed. Hecolin™ is a safe and effective anti-HEV vaccine for healthy subjects 16 years of age and older exposed to HEV genotype 4. The vaccine is now licensed in China [14]. This vaccine shows promise for outbreak interventions and control of endemic disease but several epidemiological issues need to be addressed before the vaccine is used widely in Africa. The participants in the efficacy trial used for licensing Hecolin™ were healthy adults, and there is no safety data on persons with chronic liver disease, the immunocompromised, and among children less than 16 years old. While the vaccine has also been found safe in a small number of pregnant women (n = 37) inadvertently vaccinated during a large clinical trial [138], there is a necessity for demonstrating safety in well-powered trials of pregnant women as this population should be included in outbreak response because of the high case fatality rate. While vaccine efficacy has been established for genotype 4, there are no studies in patients with genotype 1 or 2, the prominent strains in Africa. For outbreaks, it is unknown how quickly after the first injection protection will be afforded to those at risk early in the outbreak. There are no published reports on the length of protection. Still, these issues can and should be addressed quickly as there is a potential for this vaccine is be a major component of African HEV control program.

## Conclusions

Extensive data suggest that hepatitis E is a major contributor to disease and mortality across much of the African continent, with country-level variability, as expected. Still, it is challenging to make comparisons across these populations, given differing methodologies and assays used to determine HEV etiology. Despite its substantial impact on human health, HEV has, even in hyper-endemic South Asia, been neglected in recognition as a major public health problem since its identification. Given the emerging evidence that HEV could be vaccine preventable, we hope this review will shed light on a pathogen of significance across the African continent.

## Abbreviations

HEV: Hepatitis E virus; CFR: Case fatality rate; NANB: Non A, non B; HBV: Hepatitis B virus; CAR: Central African Republic; DRC: Democratic Republic of the Congo; ORF: Open reading frame; NT: Nucleotides; NA: Not available.

## Competing interests

The authors declare that they have no competing interests.

## Authors’ contributions

TFW outlined the manuscript, oversaw the development, and with J-HK wrote the manuscript. J-HK, UP, and YK searched and J-HK reviewed the literature. All authors reviewed the results, edited the manuscript, and approved the final manuscript.

## Pre-publication history

The pre-publication history for this paper can be accessed here:

http://www.biomedcentral.com/1471-2334/14/308/prepub

## Supplementary Material

Additional file 1: Table A1 Sporadic hepatitis cases caused by NANB viruses. Table A2. NANB outbreaks in Africa. Table A3. Case-fatality rates (CFRs) by NANB. Click here for file
