# Erratum to: A systematic review of the epidemiology of hepatitis E virus in Africa

**DOI:** 10.1186/s12879-017-2274-3

**Published:** 2017-03-03

**Authors:** Jong-Hoon Kim, Kenrad E. Nelson, Ursula Panzner, Yogita Kasture, Alain B. Labrique, Thomas F. Wierzba

**Affiliations:** 1 International Vaccine Institute, SNU Research Park, 1 Gwanak-ro, Gwanak-gu, Seoul 08826 Korea; 2 Department of Epidemiology, Bloomberg School of Public Health, Johns Hopkins University, 615 N. Wolfe Street, Baltimore, Maryland 21205 USA; 3 PATH, 2201 Westlake Avenue, Suite 200, Seattle, WA 98121 USA

## Erratum

In this letter, we wish to correct errors in the previously published article [1]. Although the errors do not change the main results and conclusions described in the abstract of the original article, we believe providing the correct information is important. The major correction is about the genotype distribution of HEV in Africa. In the original article, we indicated that genotype 3 is rare and less commonly found than genotype 2 while genotype 1 is the most prevalent. The correct information is, however, that genotypes 2 and 3 were identified at a similar frequency while genotype 1 was the most prevalent. This error arose because the genotypes of HEV identified in seven Nigerian adults [89] were mistaken to be 2, when their actual genotype was 3. In what follows, we revised the relevant section named “Genotype prevalence” on page 5 of the original article and the relevant table and figure (i.e., Table 5 and Fig. 2).

**Table 1:** Seroprevalence of anti-HEV antibodies in Africa. Seroprevalence varies by country and by subpopulation and studies were done under different conditions (e.g., sample size, demographics, and different diagnostic methods). Age of the sample is provided as mean (range or ± standard deviation, if available)

Country	% sero-prevalence	Sample demographics	Sample size	Year of sampling	Diagnostic methods	Source
Burkina Faso	19.1	Blood donors	178	2010-12	IgG	[29]
	11.6	Pregnant women	189	2010-12	IgG	[29]
Burundi	14.0	Adults without chronic liver disease, 44.7 yrs old (±13.5)	129	1986	Total Ig	[30]
Cameroon	14.2	HIV-infected adults, 38.1 yrs old (±11.3)and	289	2009-10	IgG	[32]
	2.0	HIV-infected children, 8.3 yrs old (±7.5)	100	2009-10	IgG	[32]
CAR^a^	24.2	Patients attending the center for sexually transmitted diseases	157	1995^b^	Total Ig	[33]
Djibouti	13.0	Male peacekeepers in Haiti, 31.2 yrs old	112	1998^b^	Total Ig	[42]
Egypt	84.3	Pregnant women, 24 yrs old (16-48)	2,428	1997-2003	Total Ig	[55]
	80.1	Patients with chronic liver disease, 48 yrs old (23-62)	518	2000-2	IgG	[57]
	67.6	Residents of two rural villages, 24.5 and 26.5 yrs, respectively	10,156	1997	Total Ig	[54]
	58.6	Asymptomatic pregnant women, ~33 yrs old	116	2009	IgG	[58]
	56.4	Residents of a semi-urban village, 1-67 yrs old	140	1993	Total Ig	[51]
	54.1	Four waste water treatment plant male workers, 20-60 yrs old	205	1998-9	IgG	[116]
	51.2	Waste water treatment plant workers, 47.1 yrs old	43	2011^b^	Total Ig	[60]
	50.6	Waste water treatment plant workers, 20-60 yrs old	233	2000^b^	Total Ig	[61]
	45.3	Blood donors, 18-45 yrs old	95	1998^b^	IgG	[52]
	39.6	Haemodialysis patients, 8-20 yrs old	96	1998^b^	IgG	[52]
	38.9	Healthy females, 21.8 yrs old (16-25)	95	1995	IgG	[50]
	17.2	Residents of a hamlet, 20.9 yrs old (<1-95)	1259	1992	IgG	[49]
	0.0	Healthy controls, 20–60 yrs old	96	1998-9	IgG	[116]
Gabon	14.2	Pregnant women, 24.6 yrs old (14-44)	840	2005, 2007	IgG	[73]
	0.0	Villagers, 29 yrs old (2-80)	35	1991-2	Total Ig	[72]
Ghana	45.3	Adult HIV patients, 40 yrs old (±9.6)	402	2008-10	IgG	[32]
	38.1	Pig handlers, 36.5 yrs old (12-65)	105	2009^b^	Total Ig	[77]
	34.8	Pig handlers, 32.9 yrs old (15-70)	353	2008	Total Ig	[75]
	28.7	Pregnant women, 28.9 yrs old (13-42)	157	2008	Total Ig	[78]
	4.6	Blood donors	239	2012^b^	IgG	[76]
	4.4	6-18 yr olds	803	1993	Total Ig	[74]
Madagascar	14.1	Slaughterhouse workers	427	2008-9	Total Ig	[81]
Morocco	8.5	Blood donors	200	2000-1	IgG	[85]
	2.2	men (*n* = 232) and women (*n* = 259), 27.7 yrs old (±5.9)	491	1995^b^	IgG	[84]
Nigeria	94.0	Control healthy adults (*n* = 44)	44	2008-9	Total Ig	[90]
	43.0	Health care workers	88	2008-9	Total Ig	[90]
	13.4	Healthy and sick people, 29.8 yrs old (3-72)	186	2007	Total Ig	[91]
Sierra Leone	7.6	Primary school children, 6-12 yrs old	66	1998^b^	IgG	[139]
South Africa	10.7	Urban (*n* = 407) and rural (*n* = 360) blacks, 42 yrs old (18-85)	767	1996^b^	Total Ig	[98,117]
	2.6	Medical students	227	1992	Total Ig	[97]
	1.8	Canoeists who have been regularly exposed to waste water	555	1992	Total Ig	[97]
Tanzania	6.6	Women, 32.1 yrs old (15-45)	212	1996	Total Ig	[114]
	0.2	Healthy adults, 30.3 yrs old	403	1992	Total Ig	[112]
	0.0	Women	180	1995	Total Ig	[113]
Tunisia	46.0	Healthy persons, > 60 yrs old	100	1991	IgG	[106]
	29.5	Children with chronic haematological diseases	34	1996	IgG	[106]
	28.9	Polytransfused patients; adults (*n* = 59, 34.8 yrs old [20-61]) and children (*n* = 48, 7.3 yrs old [1-15])	107	2008-9	IgG	[107]
	22.0	Healthy blood donors, < 40 yrs old	100	1996	IgG	[106]
	12.1	Pregnant women, 30.1 yrs old (17-52)	404	2008-9	IgG	[108]
	10.0	Healthy controls; blood donors (*n* = 100, 31.3 yrs old [20–58]) and children, (*n* = 60, 7.9 yrs old [1–15])	160	2008-9	IgG	[107]
	5.4	Blood donors, 32.6 yrs old (± 8.6)	687	2007-8	Total Ig	[109]
	4.3	Healthy persons, 20.7 yrs old (16-25)	1,505	2008^b^	IgG	[110]
Zambia	40.6^c^	Urban adults, 18–64 yrs old	106	1999	IgG	[115]
	16.0	Urban children, 1–15 yrs old	194	2011	IgG	[115]

^a^CAR; Central African Republic

^b^The year of the publication

^c^The original study reports 42%, but the actual figures indicate that 43 out of 106 specimens are positive; 43/106 = 0.4056

**Table 2:** Sporadic cases caused by hepatitis E virus in Africa. Proportion of sporadic hepatitis cases attributable to HEV varies by country and by subpopulation and studies were done under different conditions (e.g., sample size, demographics, and different diagnostic methods). Age of the sample is provided as mean (range or ± standard deviation, if available)

Country	% sero-positivity	Case demographics	No. of cases	Year of sampling	Diagnostic methods	Source
Chad	48.8	Acute or fulminant hepatitis patients, 4-64 yrs old	41	1993	IgM	[36]
	20.0^a^	Sporadic cases	17	1994	RT-PCR^b^	[27]
Djibouti	58.5	Acute hepatitis patients, 21.8 yrs old (2-65)	65	1992-3	IgM	[41]
Egypt	24.2	Jaundiced patients, 1-73 yrs old	202	1993	IgM	[46]
	22.2	Jaundiced children, 5 yrs old (1-11)	261	1990	IgM	[70]
	21.7	Acute hepatitis patients, 26.6 yrs old (18-60)	143	1993-4	IgM	[71]
	20.2	Acute viral hepatitis patients, 8 yrs old	287	2006-8	IgM	[62]
	17.9	Acute hepatitis patients, 15.7 (± 14.9) yrs old	235	2007-8	IgM or > = 3-fold rise in IgG	[69]
	17.2	Children with elevated level (two-fold or more) of AST and ALT	64	2006^d^	IgM	[47]
	15.7	Acute hepatitis patients, 15.9 yrs old (1-65)	235	2007-8	IgM	[63]
	15.1	Children with acute jaundice, 6.4 yrs old (1-13)	73	1987-8	IgM	[45]
	12.5	Patients with acute hepatitis, 20.2 yrs old (4-65)	200	2001-2	IgM	[64]
	6.0	Children with minor hepatic ailments, 6 mo-10 yrs	100	2004-5	IgM	[65]
	5.0	Patients with acute on chronic liver failure, 46.4 yrs old	100	2009-10	IgM	[66]
	2.1	Acute viral hepatitis patients, 25 yrs old (2-77)	47	2002-5	IgM	[76]
	2.0	Hepatitis patients, 5.4 yrs old (1.5-15)	50	2007	RT-PCR	[48]
Ethiopia	45.6	Acute viral hepatitis patients with NANB	79	1988-91	FABA^d^	[43]
	31.8	Non-pregnant women with acute viral hepatitis, 30 yrs old	22	1988-91	FABA	[6]
	67.9	Pregnant women with acute viral hepatitis, 26 yrs old	28	1988-91	FABA	[6]
Mayotte	100.0	Patients with acute jaundice, 46 yrs old	1	2009	IgM	[82]
Nigeria	70.0	Male patients with acute hepatitis, 25-33 yrs old	10	1997-8	RT-PCR	[89]
Senegal	20.0	Patients with jaundice	30	1992^c^	IgM	[93]
	10.2	Patients with viral hepatitis	49	1993^c^	IgM	[92]
Somalia	61.1	Native Somalis and displaced Ethiopian patients with acute hepatitis, 7-90 yrs old	36	1992-3	IgM	[96]
Sudan	5.4	Patients with fulminant hepatic failure, 38 yrs old (19-75)	37	2003-4	IgM	[103]
	59.0	Children with acute clinical jaundice, ≤14 yrs old	39	1987-8	IgM	[118]

^a^20% was extrapolated from the results of RT-PCR of 5 samples out of total 17 cases

^b^Reverse transcription polymerase chain reaction

^c^The year of the publication

^d^FABA; fluorescent antibody blocking assay, which is claimed to detect acute infection, not but past infection

**Table 5:** Genotype distribution from African HEVs

Genotype	Country	Year of sampling	Sample	RNA region tested	Source
1	CAR^a^	2002	One fecal sample from an outbreak	NA^b^	[34]
	Chad	1984	A patient with hepatitis E	Complete genome	[28]
		2004	Five isolates from an outbreak	ORF^c^2 (363 nt^d^)	[35]
	Egypt	1993	Acute hepatitis patients	ORF1 (location: 55-320)	[46]
		2006-8	Acute hepatitis patients	ORF1	[62]
		2012^e^	Sixteen isolates from acute hepatitis patients	ORF2 (189 nt)	[124]
	Namibia	1983	Nine isolates from an outbreak in Kavango	ORF2 (296 nt), 3 (188 nt)	[88]
	Sudan	2004	Twenty three isolates from an outbreak	ORF2 (363 nt)	[35]
	Uganda	2007	Internally displaced persons camp	NA	[123]
		2008	Twenty four isolates from an outbreak	NA	[119]
2	CAR	2002	Three fecal samples from an outbreak	NA	[34]
	Chad	2004	Four isolates from an outbreak	ORF2 (363 nt)	[35]
	Namibia	1995	Four isolates from NANB outbreak in Rundu	ORF2 (451 nt near 3'-end)	[87]
3	Nigeria	2000^e^	Ten adult acute hepatitis patients	ORF1, 2 (3'-end)	[89]
	Egypt	2007	One 9 year-old acute hepatitis patient	ORF1, 2, 2/3	[48]
	Mayotte	2009	One French acute hepatitis patient (46 yr old)	ORF2 (288 nt)	[82]
	Madagascar	2009	Slaughter house workers	ORF2,3 (1000 nt)	[81]

^a^CAR; Central African Republic

^b^NA; not available

^c^ORF; open reading frame

^d^nt; nucleotides

^e^Publication year

**Fig. 2:**
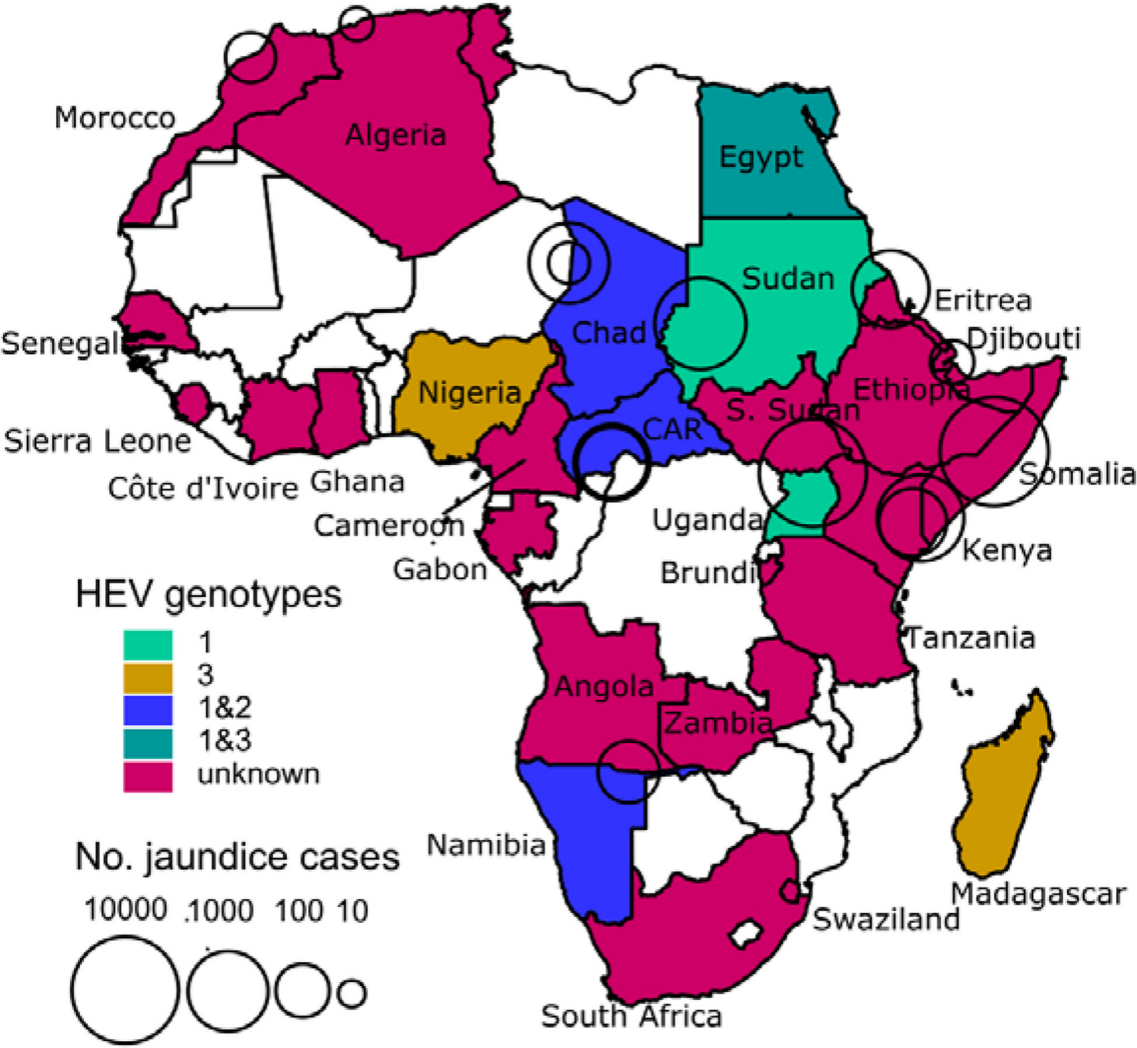
Map of Africa. Colored areas represent countries where HEV is endemic at least for some subpopulations or sporadic HEV cases or outbreaks have been detected. Circles indicate HEV outbreaks with centers and areas indicating the location and outbreak size, respectively. Different colors represent different genotypes. White areas indicate countries where no data is available

## Genotype prevalence

Data on the genotypes of circulating HEV’s are available for 9 countries (16 studies). Table 5 presents a summary sorted by genotype and also provides characteristics of the sample, genomic regions tested. Genotype 1 seems to be most prevalent as it was found in Central African Republic [34], Sudan [35], Chad [28, 35], Egypt [46, 62, 124], and Namibia [88] followed by genotype 2 and 3, of which both were observed at a similar frequency. Genotype 2 was found in Central African Republic [34], Chad [35], and Namibia [87]. Genotype 3 was observed in one Egyptian child [48], one acute hepatitis patient in Mayotte (originally from France) [82], seven Nigerian adults with acute hepatitis E [89], and slaughter house workers in Madagascar [81]. Genotype prevalence can differ in neighboring countries as was demonstrated by one study in Sudan and Chad where genotype 1 was more common in Sudan and genotype 2 was more common in Chad [35]. Figure 2 shows a map of Africa where countries in which HEV infections were observed are differently colored according to HEV genotype.

We corrected additional minor errors in Tables 1 and 2 although these corrections do not cause any changes in the main text. We have made three revisions to Table 1 of the original article:

- The seroprevalence of a Zambian population were 42% and 16%, which should be 40.6% and 16.0%, respectively [115]
- The sample size, (*n* = 402), in the description of the study conducted in Ghana (the first row of Ghana) was removed to avoid duplication
- The study of HEV in Sierra Leone was mistaken to be omitted in the original article with no reference included. It is now included in the revised Table 1 with the full reference [139]

The order of table cells was rearranged for Egyptian data by descending seroprevalence to make it consistent across countries. For Table 2, some of decimal points appear as middle dots in the original article, which were revised to be the same as other decimal points (i.e., periods) in the revised Table 2.

139. Hodges M, Sanders E, Aitken C. Seroprevalence of hepatitis markers; HAV, HBV, HCV and HEV amongst primary school children in Freetown, Sierra Leone. West Afr J Med. 1998; 17(1): 36-7.
