# Supplementary material for: A systematic review of the epidemiology of hepatitis E virus in Africa
Source: BMC Infect Dis. 2014 Jun 5;14:308. doi: 10.1186/1471-2334-14-308 (PMC4055251; doi:10.1186/1471-2334-14-308)
Supplement: Additional file 1: Table A1 — Sporadic hepatitis cases caused by NANB viruses. Table A2. NANB outbreaks in Africa. Table A3. Case-fatality rates (CFRs) by NANB. [file 1471-2334-14-308-S1.docx]

## Appendix

**Table A1. Sporadic hepatitis cases caused by NANB viruses**

| Country | % sero-  positivity | Case demographics | No. of  cases | Year | Source |
| --- | --- | --- | --- | --- | --- |
| Egypt | 50.0 | Acute hepatic children, 2-14 yrs old | 94 | Jan-Apr 1983 | [1] |
|  | 49·0 | Acute viral hepatitis children, 6·1 yrs old (1-13) | 104 | Feb 1987 – Jan 1988 | [[2]](#_ENREF_75) |
|  | 31·2 | Acute hepatitis patients | 141 | Jan – Apr 1983 | [[3]](#_ENREF_76) |
|  | 24·5 | Acute viral hepatitis patients, 20-40 yrs old | 200 | Mar – Aug 1986 | [[4]](#_ENREF_56) |
|  | 23·6 | Jaundice patients, 29·4 yrs old (12-50) for NANB | 110 | Dec 1980 – Jan 1982 | [[5]](#_ENREF_77) |
| Kenya | 18·0 | Acute viral hepatitis patients | 94 | Apr1982 – Sep 1983 | [[6]](#_ENREF_85) |
| Libya | 18.2 | Children with hepatitis | 22 | 1979-81 | [7] |
| Malawi | 21·2 | Acute hepatitis patients | 33 | 1980^a^ | [[8]](#_ENREF_88) |
| Morocco | 43·1 | Acute viral or fulminant hepatitis patients, 28 yrs old (14-60) | 239 | 1983 – 6 | [[9]](#_ENREF_90) |
| Nigeria | 16.0 | Acute viral hepatitis patients, 26.5 yrs old (14-53) | 200 | Jun 1977 – Dec 1979 | [10] |
| South Africa | 8·3 | Children with acute fulminant viral hepatitis | 12 | Jan 1986 – Dec 1989 | [[11]](#_ENREF_152) |
| Sudan | 73·9 | Acute hepatitis patients, 30.3 yrs old (13-80) | 119 | 1984-5, 12 months | [[12]](#_ENREF_112) |
|  | 43·8 | Acute viral hepatitis children, 6·1 yrs old (1-14) | 80 | Jan 1987 – May 1988 | [[13]](#_ENREF_113) |
| Zimbabwe | 7·8 | Acute viral hepatitis patients, 23 yrs old (2-69) | 141 | Oct 1981 – Feb 1982 | [[14]](#_ENREF_153) |

^a^Publication year

**Table A2. NANB outbreaks in Africa**

| Country | Year | No. cases (deaths) | Clinical attack rate  (population size) | Variance in clinical attack rate | | Source |
| --- | --- | --- | --- | --- | --- | --- |
|  |  |  |  | By age | By gender |  |
| Algeria | 1980 | ~ 40 (NA^a^) | NA | Mainly young adults | NA | [[15]](#_ENREF_28) |
|  | 1983 | 5 (0, 1 stillbirth) | 16·1% (n=36) | All cases were 18-30 yrs old | 3 males and 2 females (one pregnant) |  |
|  | Oct 1980 – Jan 1981 | 788 (9) |  | 90.1% (710/788) were adults | Among adults (n=710), 422 were males 288 female | [16] |
| Botswana | Jun – Dec 1985 | 273 (>4) | 1-2% (n=NA) | 90.3% (214/237) were aged 20 yrs or older | 122 (51%) males and 115 (49%) females | [[17]](#_ENREF_32) |
| Chad | Oct1983 – Apr 1984 | 38 | NA | All cases were 19-39 yrs old | NA | [18] |
| Somalia^c^ | Jan1985 – Sep 1986 | >2000 (87) | 8% among adults and 1.8% children (<15 years of age) (n=2000) | Adults accounted for 81% during the first outbreak (Jan-Mar 1985). | NA | [19] |
| Sudan^c^ | Jun – Oct 1985 | NA (63) | NA | 66% were adults 15 yrs of age | Twice as many males as females | [19] |

^a^Not available

^b^Attack rate is computed through survey of 2000 people

^c^Outbreak in refugee camps

**Table A3. Case-fatality rates (CFRs) by NANB**

| Country | Year | Case-fatality rate (n=no. cases) | | Source |
| --- | --- | --- | --- | --- |
|  |  | Pregnant female | Overall |  |
| Algeria | Oct 1980 – Jan 1981 | 100% (n=9) | NA^a^ | [[16]](#_ENREF_27) |
| Botswana | Jun – Dec 1985 | NA | >1·5% (n=273) | [[17]](#_ENREF_32) |
| Libya | 1975 | 13% (n=293) | 4.8% (n=922) | [20] |
|  | Dec1979 - Apr1981 | 4.7% (n=43) | NA | [21] |
| Morocco | 1983 – 6 | NA | 12·6% (n=103) | [[9]](#_ENREF_90) |
| Somalia | Jan1985 – Sep 1986 | 17·3% (n=NA) | 4·2 % (n=2087) | [19, 22] |
| Sudan | Jun – Oct 1985 | NA | 3·1% (n=NA) | [19] |

^a^Not available

## References

1. Zakaria S, Goldsmith RS, Zakaria MS, Kamel MA, El-Raziky EH: **The etiology of acute hepatitis in hospitalized children in Cairo, Egypt.** *Infection* 1988, **16:**277-282.

2. Hyams KC, McCarthy M, el-Shimy S, el-Samahy M, Mansour MM, Imam IZ: **Acute sporadic non-A, non-B hepatitis in a pediatric population living in Cairo, Egypt.** *Infection* 1990, **18:**273-276.

3. Zakaria S, Goldsmith RS, Kamel MA, el-Raziky EH: **The etiology of acute hepatitis in adults in Egypt.** *Trop Geogr Med* 1988, **40:**285-292.

4. Darwish MA, Shaker M, al-Kady AM: **Non-A, non-B viral hepatitis in Egypt.** *J Egypt Public Health Assoc* 1992, **67:**171-179.

5. Bassily S, Boctor FN, Farid Z, Fanous A, Yassin MY, Wallace CK: **Acute hepatitis non-A non-B in Cairo residents (a preliminary report).** *Trans R Soc Trop Med Hyg* 1983, **77:**382-383.

6. Greenfield C, Karayiannis P, Wankya BM: **Aetiology of acute sporadic hepatitis in adults in Kenya.** *J Med Virol* 1984, **14:**357-362.

7. Gebreel AO, Christie AB: **Viral hepatitis in children: a study in Libya.** *Ann Trop Paediatr* 1983, **3:**9-11.

8. Supran EM, Molyneux ME, Banatvala JE: **IgM responses to hepatitis-A virus and hepatitis-B core antigen in acute and chronic liver disease in Malawi; possible role of non-A, non-B, hepatitis.** *Trans R Soc Trop Med Hyg* 1980, **74:**389-392.

9. Rioche M, Himmich H, Cherkaoui A, Mourid A, Dubreuil P, Zahraoui M, Pillot J: **[High incidence of sporadic non-A, non-B hepatitis in Morocco: epidemiologic study].** *Bull Soc Pathol Exot* 1991, **84:**117-127.

10. Ayoola EA: **Non-A, Non-B hepatitis in Nigerians.** *East Afr Med J* 1983, **60:**688-691.

11. Friedland IR, Zuckerman M, Kala UK, Parbhoo KB: **Fulminant hepatitis in children: report of 12 cases.** *Ann Trop Paediatr* 1991, **11:**207-211.

12. Al-Arabi MA, Hyams KC, Mahgoub M, Al-Hag AA, el-Ghorab N: **Non-A, non-B hepatitis in Omdurman, Sudan.** *J Med Virol* 1987, **21:**217-222.

13. Hyams KC, Hussain MAM, Alarabi MA, Atallah NA, Eltigani A, McCarthy MC: **Acute Sproradic Hepatitis in Sudannese Children.** *J Med Virol* 1991, **33:**73-76.

14. Crocchiolo PR, Caredda F, D'Arminio Monforte A, Lencioni R, Ragni MC, Cenzuales S, Farci P, Lavarini C, Latif AS: **The aetiology of acute hepatitis in Zimbabwe.** *Trans R Soc Trop Med Hyg* 1984, **78:**514-518.

15. Nouasria B, Larouze B, Dazza MC, Gaudebout C, Saimot AG, Aouati A: **Non-A, non-B Hepatitis Epidemic in Eastern Algeria.** *Bulletin De La Societe De Pathologie Exotique* 1985, **78:**903-906.

16. Belabbes EH, Bouguermouh A, Benatallah A, Illoul G: **Epidemic non-A, non-B viral hepatitis in Algeria: strong evidence for its spreading by water.** *J Med Virol* 1985, **16:**257-263.

17. Byskov J, Wouters JS, Sathekge TJ, Swanepoel R: **An outbreak of suspected water-borne epidemic non-A non-B hepatitis in northern Botswana with a high prevalence of hepatitis B carriers and hepatitis delta markers among patients.** *Trans R Soc Trop Med Hyg* 1989, **83:**110-116.

18. Molinie C, Saliou P, Roue R, Denee JM, Farret O, Vergeau B, Vindrios J: **Acute epidemic non-A, non-B Hepatitis (NANBH): clinical study of 38 cases seen in Chad.** *J Med Virol* 1987, **21:**A30-A30.

19. Centers for Disease Control and Prevention: **Enterically transmitted non-A, non-B hepatitis--East Africa.** *MMWR Morb Mortal Wkly Rep* 1987, **36:**241-244.

20. Christie AB, Allam AA, Aref MK, Muntasser IH, El-Nageh M: **Pregnancy hepatitis in Libya.** *Lancet* 1976, **2:**827-829.

21. Gebreel AO, Dane DS: **Hepatitis in pregnancy in Libya.** *Ann Trop Med Parasitol* 1983, **77:**321-322.

22. Weisfuse I, Gove S, Kane M, Salaad A, Bradley D: **Enterically Transmitted non-A, non-B hepatitis Among Refugees In Somalia.** *Journal of Medical Virology* 1987, **21:**A30-A30.
